# Diode Laser Reduction of Symptomatic Inferior Turbinate Hypertrophy

**DOI:** 10.31729/jnma.4005

**Published:** 2018-12-31

**Authors:** Nain Bahadur Mahato, Deepak Regmi, Meera Bista

**Affiliations:** 1 Department of ENT-HNS, Kathmandu Medical College, Sinamangal, Kathmandu, Nepal

**Keywords:** *diode laser*, *fiberoptic laryngoscopy*, *inferior turbinate hypertrophy*, *VAS score*

## Abstract

**Introduction:**

Inferior turbinate hypertrophy refractory to medical management is one of the commonest problems encountered by ENT surgeons all over the world. Diode Laser turbinate reduction is a safe, minimally invasive, cost-effective procedure that helps in relieving the symptoms associated with it and can be performed on a day care basis under local anesthesia. The objective of this study is to measure the effectiveness of Diode laser in treatment of symptomatic ITH.

**Methods:**

Fifty patients with symptomatic inferior turbinate hypertrophy, age ranging between 1545 years were enrolled in the study. Symptom assessment was done with the visual analogue scale. Portable Diode laser was used. Patients were followed up after 1 week and 1 month of surgery. During each postoperative visit, symptoms were reassessed using VAS and postoperative morbidity were noted.

**Results:**

Out of fifty patients, all the patients had nasal obstruction and 42 had nasal discharge and by the end of 1 month 43 (86%) and 37 (88%) patients had relief of symptoms respectively. Excessive sneezing was found in 45 patients, 39 (86.6%) patients got benefitted. Out of 18 patients, 15 (83.3%) patients had decreased snoring at 1 month post-op. Similarly, 24 patients had headache, 20 (83.3%) patients had decrease in severity. Hyposmia was seen in 19 patients, 10 (52.6%) patients had improvement in olfaction.

**Conclusions:**

Diode laser turbinate reduction for symptomatic inferior turbinate hypertrophy is one of the safest procedures that can be done under local anesthesia on OPD basis with significant relief of symptoms and with minimal complications.

## INTRODUCTION

Inferior Turbinate Hypertrophy (ITH) is usually seen in patients with allergic, infective, vasomotor and hormonal rhinitis.^1^ A patient with ITH may present with excessive sneezing, rhinorrhea, hyposmia and headache in addition to nasal obstruction.

Chronic inflammation of the nasal mucosa leads to deposition of collagen in the submucosal tissue of the turbinates and remodelling of the turbinate bone leading to ITH.^1,2^ Initially the symptoms may be responsive to medical management such as topical decongestants, antihistamines and steroids.^3^

Surgical reduction of inferior turbinate is usually done for the cases refractory to medical treatment.^4^ The diode laser is one of the most portable and least expensive among various lasers available for turbinate reduction under local anesthesia with excellent patient acceptance.^5,6^ The objective of this study is to measure the effectiveness of Diode laser in treatment of symptomatic ITH.

## METHODS

This descriptive cross-sectional study was done in the department of ENT-HNS of Kathmandu Medical College from 1^st^ Jan 2018–30^th^ June 2018. History of nasal obstruction, nasal discharge, headache, excessive sneezing, snoring and hyposmia not responding to medical management were noted and the symptom assessment was done with the visual analogue scale (VAS) in which the patient rates his symptoms from a score of 0 to 10, score 0 being asymptomatic and 10 being the most severe symptoms. Patients with previous history of surgical interventions or nasal trauma, gross deviation of nasal septum or nasal polyposis, and malignancy of nose and paranasal sinuses, were excluded from the study. Ethical clearance was taken from the Ethical clearance committee of KMCTH. Written consent was taken from the patients to be enrolled in the study. All the patients posted for the procedure had to undergo fibreoptic laryngoscopic (FOL) examination by the consultant otolaryngologists to assess the size of inferior turbinate and to exclude nasal polyposis, septal deviation and malignancy of nose and paranasal sinuses. Fifty patients with symptomatic inferior turbinate hypertrophy, age ranging between 15–45 years were enrolled in the study and purposive sampling method was used.

Local anaesthesia (15% xylocaine spray and 2% xylocaine injection) was used simultaneously. The Portable Diode laser (wavelength of 980 nm, Fox diode laser from A.R.C) was used in contact mode with fibre diameter of 600 /vm, power of 10 to 12 W in continuous wave mode for 100–140 s. The probe of laser was inserted into the submucosal layer of inferior turbinate and multiple linear strokes along the length of turbinate were applied submucosally, inorder to avoid the mucosal injury. Patients were followed up after 1 week and 1 month of surgery. During each postoperative visit, FOL was done and the symptoms were reassessed using VAS and postoperative morbidity like pain, bleeding and crusting was noted. All the data were analysed using SPSS version 18.

## RESULTS

Out of fifty patients, all the patients had nasal obstruction and the average VAS score was 9.25 preoperatively and by the end of 1 month 43 (86%)patients had relief of symptoms and the average VAS score was 1.15. Similarly, 42 patients had nasal discharge in pre-op with average VAS score 7.25, of them 37 (88%) patients had relief of symptoms with fall in average VAS score to 0.27. Excessive sneezing was found in 45 patients with average VAS score 7.86 and at 1 month follow-up, 39 (86.6%) patients had benefitted of symptoms with average VAS score 1.12. Out of 18 patients, 15 (83.3%) patients had decreased snoring at 1 month post-op. Similarly, 24 patients had headache and by the end of 1 month, 20 (83.3%) patients had decrease in severity. Hyposmia was seen in 19 patients preoperatively, 10 (52.6%) patients had improvement in olfaction (Table 1, 2 and 3).

**Table 1:** Showing number of patients having preoperative symptoms and average VAS score.

Symptoms	Number of patients with symptoms (%)	AverageVAS score
Nasal Obstruction	50 (100%)	9.25
Nasal Discharge	42 (84%)	7.25
Sneezing	45 (90%)	7.86
Snoring	18 (36%)	3.25
Headache	24 (48%)	4.57
Hyposmia	19 (38%)	3.35

**Table 2:** Showing average VAS score in preoperative, 1^st^ and 2^nd^ postoperative period.

Symptoms	Preoperative VAS score	1^st^ postoperative VAS score (1 week)	2^nd^ postoperative VAS score (1 month)
Nasal Obstruction	9.25	2.25	1.15
Nasal Discharge	7.25	2.27	0.27
Sneezing	7.86	2.78	1.12
Snoring	3.25	1.05	0.75
Headache	4.57	1.75	0.58
Hyposmia	3.35	2.15	1.75

**Table 3:** Showing number of patients who got relief of symptoms 1 month postoperatively.

Symptoms	Number of patients having symptoms preoperatively	Number of patients who got relief 1 month postoperatively (%)
Nasal Obstruction	50	43 (86%)
Nasal Discharge	42	37 (88%)
Sneezing	45	39 (86.6%)
Snoring	18	15 (83.3%)
Headache	24	20 (83.3%)
Hyposmia	19	10 (52.6%)

In our study, only few patients had complications such as pain, bloody nasal discharge and mucosal edema in immediate postoperative period which completely resolved by the end of 1 month. Eight (16%) patients had crusting at 1^st^ postoperative week which resolved at 1 month follow-up (Table 4).

**Table 4:** Showing number of patients having complications in immediate, 1 week and 1 month postoperative period.

Complications	Immediate postoperative number (%)	1 week postoperative number (%)	1 month postoperative number (%)
Bloody Nasal Discharge	8 (16%)	2 (4%)	0
Pain	15 (30%)	1 (2%)	0
Crusting	0 (0%)	8 (16%)	0
Synechiae	0 (0%)	0 (0%)	0
Mucosal Edema	6 (12%)	1 (2%)	0

## DISCUSSION

Nasal obstruction due to inferior turbinate hypertrophy (ITH) causes significant morbidity as it affects the daily activities of the patients. The first line of treatment for ITH is medical and surgery is the treatment of choice for refractory cases. The symptom assessmentwas done with the VAS score both in pre and postoperative period. Maxwell^7^ has described VAS as easy to use, sensitive and accurate when testing differences within subject comparisons. In our study, we found that all patients had partial or complete improvement in all symptoms VAS scores at 1 week and 1 month postoperatively. Forty three (86%) out of 50 patients had relief of symptoms for nasal obstruction at 1 month follow-up. The average VAS score preoperatively was 9.25 and at 1 month follow-up it was found to be 1.15. In the initial 2–3 days following the surgery, nasal obstruction worsened in most of the patients. This may have been due to the postoperative edema and crusting, but by the end of the first week all patients had significant improvement in nasal obstruction. Similar study done by Rhee et al.^8^ in which he compared RFVTR and diode LTR, he found that after 2 months in LTR group, 87.5% of the patients showed improvement in both the severity and frequency of nasal obstruction and statistically significant improvement was observed at 1 month after treatment. Min et al.^9^ reported that in diode LTR (Laser Turbinate Reduction) group had significant improvement in nasal obstruction and significant decrease in nasal airway resistance 6 months following the surgery which is similar to our results but the follow-up duration is longer. Similarly, Volk et al.^10^ reported that diode laser turbinoplasty had a total of 73.2% of patients had improvement of nasal obstruction and only 12.2% experienced deterioration. Unlike our study, Janda et al.^11^showed that diode LTR group did not have much improvement in the symptom of nasal obstruction at 1 month follow-up, due to the postoperative edema and crusting but they had statistically significant improvement of the nasal airflow and nasal cavity volume at 6 months and 1 year respectively.

Preoperatively, 42 patients had the complaints of nasal discharge and in the first week, 8 patients complained of persistent rhinorrhea but by the end of 1 month, 37 (88%) had relief of symptoms. The relief in rhinorrhea may be attributed to destruction of highly vascular submucosa, seromucinous glands and destruction of branches of posterior nasal nerve, which plays a crucial role in sneezing and hyper-secretion. Forty five patients had excessive sneezing in pre-operative period and by the end of 1 month, 39 (86.6%) patients had relief of sneezing. This decrease in excessive sneezing might be due to destruction of posterior nasal nerve. A study by Supiyaphun et al.^12^ showed that KTP LTR produced significant reduction in rhinorrhea postoperatively. Preoperatively all of the patients had significant sneezing but by the end of the 6 month follow-up period it was found that 86.7% of the patients had got significant relief insneezing. The reason for relief of sneezing may be attributed to destruction of the branches of posterior nasal nerve. These findings are similar to other studies.^6,13^

In our study, 24 patients had headache and 19 patients had hyposmia preoperatively and at the end of 1 month, 20 (83.3%) and 10 (52.6%) patients had relief of headache and hyposmia respectively. This improvement may be because of improved nasal patency. Similarly, Parida et al.^2^ found that at the end of the 6 month follow up period 79.4% of the patients showed an improvement in headache and the relief in hyposmia was seen in 11 (47.8%) out of 23 (51.06%) patients, 6 months postoperatively. This finding is similar to other reported series.^6,8^ Rhee et al.^8^ compared the efficacy of radiofrequency and laser reduction of inferior turbinate on improvement of olfaction and found that after 8 weeks post-op, half of the sample showed significant improvement in olfaction in both the groups. Likewise, Maskell et al.^6^ reported that laser turbinate reduction produced a significant improvement in olfaction post-operatively.

In our study, we found that only 8 (16%) patients had bloody nasal discharge in the immediate postoperative period and then only 2 (4%) patients of them had persistent symptom in the 1^st^ postoperative week, ultimately none of them had the symptom at 1month follow-up. Fifteen patients had pain in immediate postoperative period which resolved at 1 month follow-up. Unlike other study, only 8 (16%) patients had crusting at 1^st^ postoperative week which resolved completely at the end of 1 month postoperative. This might be due to submucosal vaporisation of tissues without traumatising the nasal mucosa. As the nasal mucosa was not traumatised, none of the patients had synechiae formation inour study.

## CONCLUSIONS

Diode Laser turbinate reduction is a safe, minimally invasive, cost-effective procedure that helps in relieving the symptoms associated with ITH, and can be performed on a day care basis under local anaesthesia. There wasimprovement in postoperative average VAS score as well as relief of most of the symptoms at the end of the study without any serious intraoperative and postoperative complications. Submucosal coagulation and vaporisation of hypertrophied inferior turbinate caused less crusting and synechiae formation. Therefore most of the patients got relief of symptoms in earlier follow-up within 1 month.
